# Vitamin D deficiency in chronic inflammatory rheumatic diseases: results of the cardiovascular in rheumatology [CARMA] study

**DOI:** 10.1186/s13075-015-0704-4

**Published:** 2015-08-14

**Authors:** Ana Urruticoechea-Arana, María A. Martín-Martínez, Santos Castañeda, Carlos A. Sanchez Piedra, Carlos González-Juanatey, Javier Llorca, Federico Díaz-Gonzalez, Miguel A. González-Gay, on behalf of the CARMA Project Collaborative Group

**Affiliations:** Division of Rheumatology, Hospital Can Misses, Calle Corona s/n, 07800 Ibiza, Spain; Research Unit of Spanish Society of Rheumatology, Calle Marqués del Duero, 5 1°A, 28001 Madrid, Spain; Division of Rheumatology, Hospital U de la Princesa, IIS-IPrincesa, Calle Diego de León 62, 28006 Madrid, Spain; Division of Cardiology, Hospital Lucus Augusti, Lugo, 28001 Spain; Division of Epidemiology and Computational Biology, School of Medicine, University of Cantabria, CIBER Epidemiología y Salud Pública (CIBERESP), Santander, Spain; Research Unit of Spanish Society of Rheumatology, Madrid, Spain; School of Medicine, Universidad de La Laguna, Tenerife, Spain; Division of Rheumatology, Hospital Universitario de Canarias, La Laguna, Tenerife Spain; Division of Rheumatology, Hospital Universitario Marqués de Valdecilla, Santander, and Epidemiology, Genetics and Atherosclerosis Research Group on Systemic Inflammatory Diseases, Rheumatology Division, IDIVAL, Avenida de Valdecilla, s/n, 39008 Santander, Spain

## Abstract

**Introduction:**

The aim was to study the association between 25-hydroxyvitamin D (25(OH)D) levels and the clinical characteristics of patients with chronic inflammatory rheumatic diseases (CIRD).

**Methods:**

We studied a cross-section from the baseline visit of the CARMA project (CARdiovascular in rheuMAtology), a 10-year prospective study evaluating the risk of cardiovascular events in rheumatoid arthritis (RA), ankylosing spondylitis (AS) and psoriatic arthritis (PsA) patients, and non-CIRD patients who attended rheumatology outpatient clinics from 67 hospitals in Spain. Non-CIRD group was frequency matched by age with the joint distribution of the three CIRD groups included in the study. 25(OH)D deficiency was defined if 25(OH)D vitamin levels were < 20 ng/ml.

**Results:**

2.234 patients (775 RA, 738 AS and 721 PsA) and 677 non-CIRD subjects were assessed. The median (p25-p75) 25(OH)D levels were: 20.4 (14.4-29.2) ng/ml in RA, 20.9 (13.1-29.0) in AS, 20.0 (14.0-28.8) in PsA, and 24.8 (18.4-32.6) ng/ml in non-CIRD patients. We detected 25(OH)D deficiency in 40.5 % RA, 39.7 % AS, 40.9 % PsA and 26.7 % non-CIRD controls (p < 0.001). A statistically significant positive association between RA and 25(OH)D deficiency was found (adjusted (adj.) OR = 1.46; 95 % CI = 1.09-1.96); p = 0.012. This positive association did not reach statistical significance for AS (adj. OR 1.23; 95 % CI = 0.85-1.80) and PsA (adj. OR 1.32; 95 % CI = 0.94-1.84). When the parameters of disease activity, severity or functional impairment were assessed, a marginally significant association between 25(OH)D deficiency and ACPA positivity in RA patients (adj. OR = 1.45; 95 % CI = 0.99-2.12; p = 0.056), and between 25(OH)D deficiency and BASFI in AS patients (adj. OR = 1.08; 95 % CI = 0.99-1.17); p = 0.07) was also found.

**Conclusions:**

Patients with RA show an increased risk of having 25(OH)D deficiency compared to non-CIRD controls.

**Electronic supplementary material:**

The online version of this article (doi:10.1186/s13075-015-0704-4) contains supplementary material, which is available to authorized users.

## Introduction

Vitamin D has raised great interest in recent decades due to its multiple physiological functions, a including significant role in the regulation of the immune system [1–7]. Vitamin D deficiency is an extremely common health problem that affects up to 50 % of the general population during winter months in the Northern hemisphere [8]. Several studies have pointed out a potential association between vitamin D deficiency and cancer, some chronic infections, cardiovascular mortality and increased risk of some autoimmune diseases [8–13], such as type I diabetes mellitus [10], multiple sclerosis [10], systemic lupus erythematosus (SLE) [9, 11, 12] and rheumatoid arthritis (RA) [9, 13]. In this regard, some authors have reported an inverse relationship between serum levels of 25-hydroxyvitamin D (25(OH)D) and disease activity or functional impairment in patients with RA or early arthritis [14–21]. In a recent study, vitamin D deficiency was found in 30 % of RA patients [8]. Most studies assessing serum 25(OH)D levels in patients with chronic inflammatory rheumatic diseases (CIRD) were focused on patients with RA [9, 13–15, 17–20]. However, fewer studies have analyzed the presence of 25(OH)D deficiency in other CIRD such as ankylosing spondylitis (AS) or psoriatic arthritis (PsA) [21–24].

The aim of this study was to assess 25(OH)D levels in a cohort of Spanish patients with CIRD that included patients with RA, AS, PsA and non-CIRD, who were attending rheumatology outpatient clinics, and to determine the potential relationship between 25(OH)D levels and clinical characteristics of every disease included in the group of patients with CIRD.

## Methods

### Study design

Cross-sectional analysis from the baseline visit of the project, CARdiovascular in rheuMAtology (CARMA), a 10-year prospective cohort study designed to determine the cardiovascular mortality risk in patients with CIRD compared to a cohort of patients without inflammatory pathological disease [25]. Information on this cohort has recently been reported. The institutional review board of each center approved the study and all patients signed an informed consent agreement.

### Patients and controls

Based on the information from the Spanish Society of Rheumatology (SER), all the Spanish public hospitals (university and general community hospitals) that have rheumatology units were invited to take part in the present study. Finally, 67 (63.2 %) of the 106 centers agreed to participate in the study. They recruited 2,986 patients who attended rheumatology outpatient clinics from July 2010 to January 2012. Seventy-five patients declined the invitation. Therefore, 2,911 patients over 18 years old were included in the study. They were split into two different cohorts according to CIRD exposition. The CIRD patients consisted of 775 patients diagnosed with RA (1987 American College of Rheumatology (ACR) classification criteria) [26], 738 diagnosed with AS (modified New York criteria) [27] and 721 patients with PsA (Moll and Wright criteria) [28]. The control (non-CIRD) group included 677 patients without inflammatory rheumatic disease. The non-CIRD group was frequency-matched by age with the joint distribution of the three CIRD groups. Therefore, its distribution approximates the joint age distribution of the three CIRD. To see the list of participating centers, we advise readers to see the Acknowledgements section.

### Variables and operative definitions

Both cohorts were evaluated following international protocols including standardized definitions and validated questionnaires. All patients included were continuously and systematically evaluated online and, to verify the quality of the information, an in situ monitoring data assessment was performed in 15 % of patients randomly selected.

The primary endpoint was the presence of 25(OH)D deficiency defined as 25(OH)D levels below 20 ng/ml. The variable sunshine hours per month and province (geographical area in which the hospital is located) was established considering the hours of sunlight in the period of time between 60 and 90 days prior to the visit of inclusion of each patient. For this purpose we used the information published by the Spanish Meteorological Agency (SMA) [29]. When information on sunshine hours of the month and year was not available, we used the mean value of sunshine hours of the last 5 years in which information on sunshine hours in the same period of time and site was available. The 25(OH)D analysis was locally performed according to the methodology and reproducibility level of each institution.

Other variables analyzed were: (1) obesity (BMI ≥30, kg/m^2^) and main physical activity during working hours (low activity: sitting most of the time; moderate activity: standing most of the time and with little movement or effort; intense activity: walking most of the time or performing tasks that require high physical activity); (2) disease characteristics and parameters of disease activity: rheumatoid factor (RF), anti-cyclic citrullinated peptide antibodies (ACPA), HLA-B27 positivity, erythrocyte sedimentation rate (ESR) (mm/1^st^ h), ultra-sensitive C-reactive protein (CRP) (mg/l), disease activity score including 28 joints-erythrocyte sedimentation rate (DAS28-ESR), health assessment questionnaire (HAQ (0–3)), Bath ankylosing spondylitis disease activity index (BASDAI (0–10)) and Bath ankylosing spondylitis functional index (BASFI (0–10)); (3) sociodemographic variables, and (4) other factors: disease severity, duration of the disease and therapies administered including calcium and vitamin D supplementation.

### Statistical analysis

Numerical variables with a normal distribution were expressed as mean and standard deviation. Variables not normally distributed were shown as median and interquartile range (IQR, percentile (p)25 − p75). Absolute and relative frequencies of qualitative variables were calculated. We performed analysis of the main demographic and clinical variables stratified by type of disease. Stratified analysis of 25(OH)D deficiency (<20 ng/ml) was performed for each group of patients according to sociodemographic characteristics and clinical factors, using the Student *t* test or the Mann-Whitney *U* test. Qualitative variables were assessed using the Chi-square test, Yates correction, or Fisher test using 2 × 2 tables.

To study the association between 25(OH)D deficiency and CIRD, logistic regression models were constructed by calculating the odds ratios (OR) with 95 % CI and adjusting for potential confounding factors. In this regard, an adjusted model for sunshine hours was performed. It was carried out considering for this purpose the period of time between 60 and 90 days prior to the baseline visit blood test, which included assessment of 25(OH)D levels. To reduce variability in the methods of measurement of 25(OH)D from each participating hospital, the mixed logistic regression models were constructed with robust variance estimators using the hospital as cluster variable estimation. The same procedure was carried out to identify specific features of each disease. The selection of independent variables in the multivariate models was based on clinical judgment and those with a *p*-value <0.20 in the bivariate analysis. In all models of logistic regression, the independent variables were adjusted for the other variables in the model.

Data management and statistical analysis were centralized at the Research Unit of the SER following a pre-established analysis plan. All the analyses were performed using the SPSS 21.0 statistical program. Statistical significance was assumed at *p* <0.05.

## Results

### Sociodemographic and clinical characteristics

Demographic and clinical characteristics of patients included in this study are summarized in Table 1. There was a predominance of women in the group with RA, whereas most AS patients were men. Sex distribution was similar in the group with PsA. The mean age in patients with RA was higher than in patients with other CIRD. Although patients with AS were younger, the duration of disease was longer than in the other groups. The frequency of obesity was higher among PsA patients, whereas it was lower among controls, despite being the group that included more sedentary individuals (*p* <0.001). Smoking history was more commonly observed in those with AS.

**Table 1:** Sociodemographic characteristics, 25(OH)D levels and clinical characteristics of the population included in the study

	Rheumatoid arthritis	Ankylosing spondylitis	Psoriatic arthritis	Controls
	(n = 775)	(n = 738)	(n = 721)	(n = 677)
Age at inclusion, years, mean (SD)	57.1 (12.3)	48.1 (11.7)	51.8 (12.0)	54.0 (12.4)
Age at the beginning of disease, years, mean (SD)	45.8 (13.4)	29.7 (11.8)	39.5 (13.3)	48.5 (12.4)
Female sex, n (%)	581 (75.0)	200 (27.1)	327 (45.4)	437 (64.5)
Educational level, n (%)				
Elementary	467 (60.9)	318 (43.3)	331 (46.3)	229 (34.1)
University /secondary	300 (39.1)	416 (56.7)	383 (53.7)	443 (65.9)
Caucasian Race, n (%)	747 (96.6)	723 (98.0)	712 (98.9)	668 (98.7)
Others	26 (3.4)	15 (2.0)	8 (1.1)	9 (1.3)
Main activity, n (%)				
Sedentary	236 (35.0)	263 (39.3)	253 (38.9)	291 (46.3)
Moderate	290 (43.0)	238 (35.5)	241 (37.1)	207 (32.9)
Active with displacement	148 (22.0)	169 (25.2)	156 (24.0)	131 (20.8)
BMI, kg/m2, mean (SD)	26.9 (4.8)	27.4 (4.4)	28.2 (4.7)	26.7 (4.4)
Obesity (BMI ≥30), n (%)	180 (23.2)	186 (25.2)	209 (29.1)	147 (21.8)
Smoking status, n (%)				
Current smokers	189 (24.4)	254 (34.4)	157 (21.8)	143 (21.2)
Past smokers	202 (26.1)	240 (32.5)	227 (31.5)	176 (26.0)
Never smokers	384 (49.5)	244 (33.1)	337 (46.7)	357 (52.8)
25 (OH) D				
25(OH)D (ng/ml), median (p25 − p75)	20.4 (14.4–29.2)	20.9 (13.1–29.0)	20.0 (14.0–28.8)	24.8 (18.4–32.6)
25(OH)D deficiency, n (%)	314 (40.5)	293 (39.7)	295 (40.9)	181 (26.7)
Sunshine hours/month*, median (p25 − p75)	162 (122–219)	165 (136–233)	178 (128–235)	301 (202–345)
Disease duration, years, median (p25 − p75)	8.0 (3.0–14.0)	15.0 (8.0–26.0)	9.0 (4.0–16.0)	2.0 (0.0–6.0)
RF-positive, n (%)	528 (68.1)	--	--	--
ACPA-positive, n (%)	482 (62.2)	--	--	--
DAS28-ESR, mean (SD)	3.2 (1.2)	--	3.0 (1.3)	--
HAQ (0–3), median (p25 − p75)	0.5 (0.1–1.1)	--	0.4 (0.0–0.9)	--
ESR (mm/h), median (p25 − p75)	17.0 (9.0–29.0)	10.0 (6.0–21.0)	12.0 (6.0–21.0)	10.0 (5.0–18)
CRP (mg/l), median (p25 − p75)	3.1 (1.2–8.0)	3.6 (1.6–8.9)	2.9 (1.4–6.1)	1.9 (1.3–3.3)
BASDAI (0–10), median (p25 − p75)	--	3.5 (1.7–5.3)	--	--
BASFI (0–10), median (p25 − p75)	--	3.1 (1.3–5.2)	--	--
HLA-B27, n (%)	--	561 (76)	--	--
Erosions (RA), n (%)	352 (45.4)	--	--	--
Biologic therapy (% ever treated), n (%)	313 (40.4)	349 (47.4)	300 (41.7)	--
Vitamin D, n (%)	325 (41.9)	82 (11.1)	114 (15.8)	138 (20.4)
Calcium supplements, n (%)	328 (42.3)	68 (9.2)	105 (14.6)	122 (18.0)

25(OH)D deficiency is defined as 25(OH)D <20 ng/ml. *Hours of sunshine per month considering the period of time between 60 and 90 days prior to the baseline visit (blood test to determine the levels of 25(OH)D was performed at the baseline visit). *BMI* body mass index, *25(OH)D* 25-hydroxyvitamin D, *p25 − p75* 25^th^ to 75^th^ percentile, *RF* rheumatoid factor, *ACPA* anti-cyclic citrullinated peptide antibodies, *DAS28-ESR*, disease activity score using 28 joints-erythrocyte sedimentation rate, *HAQ (0–3)*: health assessment questionnaire, *ESR* erythrocyte sedimentation rate, *CRP* C-reactive protein, *BASDAI (0–10)* Bath ankylosing spondylitis disease activity index, *BASFI (0–10)* Bath ankylosing spondylitis functional index, *HLA-B27* histocompatibility antigen HLA-B27, *RA* rheumatoid arthritis

It is worth noting that a great majority of patients with CIRD included in the study had low disease activity at time of recruitment. In this regard, CRP and ESR levels in RA, AS and PsA patients were remarkably low at the time of inclusion in the study, as well as the functionality scores (HAQ and BASFI, respectively).

Individuals without CIRD (controls) had the following rheumatic diseases: osteoarthritis (30 %), osteoporosis (15.2 %), soft tissue disorders (18.8 %) and other non-inflammatory diseases (36 %). The distribution at recruitment of individuals with CIRD per hospital was uniform throughout the year 2011, whereas the recruitment of patients without CIRD occurred mainly between October and November 2011 (Additional file 1: Table S1).

### 25(OH)D levels and CIRD

Patients with CIRD had lower 25(OH)D levels than those from the non-CIRD controls (Table 1). The median (p25 − p75) 25(OH)D levels were: 20.4 (14.4 − 29.2)] ng/ml in RA, 20.9 (13.1 − 29.00 ng/ml in AS, 20.0 (14.0 − 28.8) ng/ml in PsA and 24.8 (18.4 − 32.6) ng/ml in non-CIRD individuals. Globally, 25(OH)D deficiency was detected in 40.5 % of patients with RA, 39.7 % of patients with AS, 40.9 % of patients with PsA and 26.7 % of individuals with non-CIRD (*p* <0.001). The median of sunshine hours in the group of non-CIRD controls was higher than in the three groups of patients with CIRD (Table 2).

Among the variables related to activity and severity of inflammatory diseases, ACPA-positive RA patients had a higher frequency of 25(OH)D deficiency (66.9 %). It was also the case for AS, with higher values of BASDAI and BASFI in 25(OH)D-deficient patients (*p* <0.05 in both cases) (Table 2).

### Multivariate analysis

Multivariate analysis (Table 3) disclosed a positive association with 25(OH)D deficiency in the patients with CIRD when compared with the non-CIRD subjects. This association with 25(OH)D deficiency was statistically significant in the group of patients with RA (adjusted (adj.) OR = 1.46; 95 % CI = 1.09, 1.96); *p* = 0.012. However, the positive association was not statistically significant for AS (adj. OR = 1.23; 95 % CI = 0.85, 1.80) and PsA (adj. OR = 1.32; 95 % CI = 0.94, 1.84). Women with RA had significantly higher risk of 25(OH)D deficiency than men (*p* <0.01). Likewise, obese RA and PsA patients (BMI ≥30) had higher risk of 25(OH)D deficiency

**Table 2:** Bivariate analysis according to each specific entity and the occurrence of 25(OH)D deficiency (25(OH)D <20 ng/ml)

	All				Rheumatoid arthritis			Ankylosing spondylitis			Psoriatic arthritis			Controls		
Variables	Total	25OHD <20	25OHD ≥20	*p*	25OHD <20	25OHD ≥20	*p*	25OHD <20	25OHD ≥20	*p*	25OHD <20	25OHD ≥20	*p*	25OHD <20	25OHD ≥20	*p*
Female, n (%)	1545 (53.1)	554 (51.2)	796 (55.0)	0.054	220 (70.1)	280 (79.5)	0.005	92 (31.4)	81 (23.9)	0.035	127 (43.1)	162 (48.1)	0.206	115 (63.5)	273 (65.2)	0.703
Age beginning disease, years, mean (SD)	40.5 (14.5)	40.4 (14.5)	41.4 (14.7)	0.079	45.8 (13)	46.1 (13.6)	0.743	30.1 (11.7)	29.6 (11.8)	0.620	39.2 (13.1)	39.4 (13.3)	0.846	49.4 (12.6)	48.5 (12.2)	0.406
Disease duration, years, mean (SD)	11.03 (10.4)	11.5 (10.2)	10.7 (10.5)	0.005	10.1 (8.5)	10.4 (9.1)	0.782	17.6 (11.9)	17.72 (12.1)	0.950	11.2 (8.4)	11.4 (9.3)	0.983	4.4 (6.9)	4.5 (6.2)	0.321
Educational level, n (%)																
Elementary	1693 (58.6)	646 (60.4)	819 (56.8)	0.071	212 (68.6)	242 (68.9)	0.926	176 (60.7)	182 (53.8)	0.84	173 (59.5)	200 (59.5)	0.985	85 (47.5)	195 (46.9)	0.891
University/secondary	1194 (41.4)	423 (39.6)	622 (43.2)		97 (31.4)	109 (31.1)		114 (39.3)	156 (46.2)		118 (40.5)	136 (40.5)		94 (52.5)	221 (53.1)	
Smoking status, n (%)																
Current smokers	743 (25.5)	303 (28.0)	335 (23.2)		83 (24.4)	76 (21.6)		107 (36.5)	113 (33.3)		59 (20.0)	73 (21.6)		54 (30.0)	73 (17.4)	
Past smokers	845 (29.0)	332 (30.7)	407 (28.1)	0.001	86 (27.4)	92 (26.1)	0.226	95 (32.4)	107 (31.6)	0.530	101 (34.2)	104 (30.9)	0.650	50 (27.8)	104 (24.8)	<0.001
Never smokers	1322 (45.5)	447 (41.3)	705 (48.7)		145 (46.2)	184 (52.3)		91 (31.1)	119 (35.1)		135 (45.8)	160 (47.5)		76 (42.2)	242 (57.8)	
Obesity (BMI ≥30), n (%)	2184 (75.0)	327 (30.2)	308 (21.3)	0.001	88 (28.0)	71 (20.2)	0.018	83 (28.3)	75 (22.1)	0.072	102 (34.7)	89 (26.5)	0.025	54 (30.0)	73 (17.5)	0.001
Main physical activity, n (%)																
Sedentary	1043 (39.8)	380 (39.3)	553 (40.8)		92 (34.3)	113 (36.1)		103 (39.6)	120 (38.7)		110 (41.5)	119 (39.1)		75 (42.9)	181 (47.6)	
Moderate	976 (37.2)	363 (37.5)	472 (36.1)	0.734	113 (42.2)	136 (43.5)	0.669	90 (34.6)	109 (35.2)	0.976	100 (37.7)	107 (35.2)	0.386	60 (34.3)	120 (31.6)	0.576
Active with displacement	604 (23.0)	225 (23.2)	302 (23.1)		63 (23.5)	64 (20.4)		67 (25.8)	81 (26.1)		55 (20.8)	78 (25.7)		40 (22.9)	79 (20.8)	
Sunshine hours /month*, median (p25 − p75)	189 (138–269)	161 (122–225)	210 (159–320)	<0.001	156 (101–193)	184 (141–234)	<0.001	161 (122–202)	200 (157–251)	<0.001	159 (103–200)	201 (150–263)	<0.001	252 (162–345)	303 (202–345)	0.031
Clinical characteristics																
ESR (mm/h), median (p25 − p75)	-	-	-	-	18.0 (9.0–28.0)	16.0 (9.0–30.0)	0.631	10.0 (6.0–23.2)	11.0 (6.0–19.0)	0.691	12.0 (5.0–21.0)	12.0 (6.0–21.0)	0.471	11.0 (5.0–18.0)	10.0 (5.0–18.0)	0.189
CRP (mg/l), median (p25 − p75)	-	-	-	-	3.1 (1.4–8.0)	3.0 (1.0–7.3)	0.543	4.1 (1.5–10.6)	3.2 (1.6–7.6)	0.333	3.0 (1.4– 6.1)	2.9 (1.3–6.2)	0.449	2.0 (0.9–4.1)	1.9 (1.0–3.2)	0.358
RF positive, n (%)	-	-	-	-	252 (80.3)	264 (75.0)	0.105	-	-	-	-	-	-	-	-	-
ACPA positive, n (%)	-	-	-	-	210 (66.9)	205 (58.2)	0.022	-	-	-	-	-	-	-	-	-
Erosions, n (%)	-	-	-	-	143 (45.5)	169 (48.0)	0.524	-	-	-	79 (26.8)	92 (27.3)	0.883	-	-	-
DAS28-ESR, median (p25 − p75)	-	-	-	-	3.0 (2.2–3.8)	3.0 (2.3–4.0)	0.937	-	-	-	2.8 (1.9–3.9)	2.9 (2.0–3.9)	0.807	-	-	-
HAQ (0–3), median (p25 − p75)	-	-	-	-	0.5 (0.1–1.1)	0.6 (0.1–1.2)	0.193	-	-	-	0.2 (0.0–1.0)	0.3 (0.0–1.9)	0.601	-	-	-
HLA-B27, n (%)	-	-	-	-	-	-	-	218 (74.4)	275 (81.1)	0.042		-	-	-	-	-
BASDAI (0–10), median (p25 − p75)	-	-	-	-	-	-	-	3.8 (1.8–5.3)	3.3 (1.8–5.0)	0.042	-	-	-	-	-	-
BASFI (0–10), median (p25 − p75)	-	-	-	-	-	-	-	3.7 (1.6–5.8)	2.7 (1.2–4.7)	0.018	-	-	-	-	-	-
Treatment																
Biologic DMARD*, n* (%)	-	-	-	-	123 (39.2)	150 (42.6)	0.367	145 (49.5)	158 (46.6)	0.470	132 (44.7)	142 (42.1)	0.509	-	-	-
Vitamin D, n (%)	659 (22.6)	198 (18.3)	392 (27.1)	<0.001	120 (38.2)	166 (47.2)	0.020	23 (7.8)	53 (15.6)	0.003	32 (10.8)	68 (20.2)	0.001	23 (12.7)	105 (25.1)	<0.001
Calcium supplements, n (%)	623 (21.4)	191 (17.6)	353 (24.4)	<0.001	118 (37.6)	163 (46.3)	0.023	20 (6.8)	42 (12.4)	0.019	33 (11.2)	56 (16.6)	0.050	20 (11.0)	92 (22.0)	0.002

*Hours of sunshine per month considering the period of time between 60 and 90 days prior to the baseline visit (blood test to determine the levels of 25(OH)D was performed at the baseline visit). *25(OH)D* 25-hydroxyvitamin D, *BMI* body mass index, *p25 − p75* 25^th^ to 75^th^ percentile, *ESR* erythrocyte sedimentation rate, *CRP* C-reactive protein, *RF* rheumatoid factor, *ACPA* anti-cyclic citrullinated peptide antibodies, *DAS28-ESR*, disease activity score using 28 joints-erythrocyte sedimentation rate, *HAQ (0–3)*: health assessment questionnaire, *HLA-B27* histocompatibility antigen HLA-B27, *BASDAI (0–10)* Bath ankylosing spondylitis disease activity index, *BASFI (0–10)* Bath ankylosing spondylitis functional index, *DMARD* disease-modifying anti-rheumatic drugs

**Table 3:** Multivariate analysis of 25(OH)D deficiency (25(OH)D levels <20 ng/ml) in patients with chronic inflammatory rheumatic diseases

	All			Rheumatoid arthritis			Ankylosing spondylitis			Psoriatic arthritis		
Variables	Crude OR	Adjusted OR	*p*	Crude OR	Adjusted OR	*p*	Crude OR	Adjusted OR	*p*	Crude OR	Adjusted OR	*p*
	(95 % CI)	(95 % CI)		(95 % CI)	(95 % CI)		(95 % CI)	(95 % CI)		(95 % CI)	(95 % CI)	
Kind of disease (ref. controls)												
Rheumatoid arthritis	2.07 (1.53, 2.79)	1.46 (1.09, 1.96)	0.012	--	--		--	--		--	--	
Ankylosing spondylitis	2.00 (1.46, 2.74)	1.23 (0.85, 1.80)	0.273	--	--		--	--		--	--	
Psoriatic arthritis	2.03 (1.46, 2.81)	1.32 (0.94, 1.84)	0.110	--	--		--	--		--	--	
Age beginning disease	1.00 (0.99, 1.00)	1.00 (0.99, 1.01)	0.985	1.00 (0.99, 1.01)	0.99 (0.98, 1.01)	0.324	1.00 (0.99, 1.02)	1.00 (0.98, 1.02)	0.715	1.00 (0.99, 1.01)	1.00 (0.98, 1.01)	0.688
Sex (ref. male)	0.86 (0.74, 0.99)	1.06 (0.88, 1.27)	0.561	0.60 (0.43, 0.85)	0.64 (0.42, 0.97)	0.037	1.46 (1.07, 1.99)	1.54 (1.10, 2.17)	0.013	0.82 (0.61, 1.10)	0.95 (0.66, 1.37)	0.781
Disease duration, years	1.01 (1.00, 1.02)	1.00 (0.99, 1.01)	0.894	1.00 (0.98, 1.01)	1.00 (0.98, 1.02)	0.971	1.00 (0.98, 1.01)	1.00 (0.98, 1.02)	0.841	0.998 (0.980, 1.015)	1.00 (0.98, 1.01)	0.581
Educational level (ref. elementary)												
University/secondary	0.86 (0.67, 1.11)	0.99 (0.77, 1.29)	0.963	1.02 (0.71, 1.45)	1.17 (0.69, 1.72)	0.430	0.76 (0.52, 1.10)	0.81 (0.55, 1.19)	0.280	1.00 (0.67, 1.50)	0.94 (0.62, 1.41)	0.765
Smoking status (ref. current smokers)												
Past smokers	0.90 (0.74, 1.10)	0.91 (0.73, 1.13)	0.387	0.86 (0.58, 1.26)	0.80 (0.49, 1.29)	0.354	0.94 (0.62, 1.41)	1.11 (0.72, 1.70)	0.639	1.20 (0.77, 1.88)	1.20 (0.72, 1.99)	0.480
Never smokers	0.70 (0.57, 0.86)	0.73 (0.58, 0.91)	0.005	0.72 (0.51, 1.02)	0.74 (0.50, 1.11)	0.144	0.81 (0.53, 1.22)	0.84 (0.56, 1.26)	0.404	1.04 (0.66, 1.64)	1.12 (0.67, 1.87)	0.662
Obesity (BMI ≥30. kg/m^2^)	1.60 (1.32, 1.94)	1.96 (1.28, 1.90)	<0.001	1.54 (1.09, 2.17)	1.76 (1.18, 2.62)	0.006	1.39 (0.93, 2.09)	1.20 (0.78, 1.86)	0.408	1.47 (1.04, 2.09)	1.41 (0.98, 2.05)	0.067
Sunshine hours/month*	0.99 (0.99, 0.99)	0.99 (0.99, 0.99)	<0.001	0.99 (0.99, 0.99)	0.99 (0.99, 0.99)	0.001	0.99 (0.99, 0.99)	0.99 (0.99, 0.99)	0.001	0.99 (0.99, 0.99)	0.99 (0.99, 0.99)	<0.001
HAQ (0–3)				0.86 (0.69, 1.07)	0.90 (0.71, 1.14)	0.384	--	--		--	--	
ACPA-positive	--	--		1.45 (1.00, 2.09)	1.45 (0.99, 2.12)	0.056	--	--		--	--	
HLA-B27	--	--		--	--		0.68 (0.50, 0.92)	0.70 (0.48, 1.02)	0.062	--	--	
BASFI (0–10)	--	--		--	--		1.10 (1.02, 1.18)	1.08 (0.99, 1.17)	0.070	--	--	
Vitamin D therapy	0.60 (0.47, 0.77)	0.57 (0.43, 0.76)	<0.001	0.69 (0.48, 0.99)	0.78 (0.52, 1.18)	0.245	0.46 (0.26, 0.81)	0.43 (0.26, 0.81)	0.008	0.48 (0.28, 0.82)	0.54 (0.29, 1.01)	0.053

*Hours of sunshine per month considering the period of time between 60 and 90 days prior to the baseline visit (blood test to determine the levels of 25(OH)D was performed at the baseline visit). *25(OH)D* 25-hydroxyvitamin D, *OR* odds ratio, *BMI* body mass index, *HAQ (0–3)*: health assessment questionnaire, *ACPA* anti-cyclic citrullinated peptide antibodies, *HLA-B27* histocompatibility antigen HLA-B27, *BASFI (0–10)* Bath ankylosing spondylitis functional index

When the parameters of disease activity, severity or functional impairment were assessed, a marginally significant association between 25(OH)D deficiency and ACPA-positivity in RA patients (adj. OR = 1.45; 95 % CI = 0.99, 2.12; *p* = 0.056), and between 25(OH)D deficiency and BASFI in AS patients (adj. OR = 1.08; 95 % CI = 0.99, 1.17; p = 0.07) was also found (Table 3).

## Discussion

Our results show that Spanish patients with RA attending rheumatology outpatient clinics have 25(OH)D deficiency. This baseline result is from a cohort of patients that has been followed prospectively to determine the cardiovascular outcome. To establish comparisons, we also assessed baseline 25(OH)D levels in non-CIRD controls attending the same rheumatology outpatient clinics [25].

Vitamin D plays an important role in the immune regulation [2]. Vitamin D deficiency has been observed in some autoimmune diseases, in particular in SLE [11, 12, 30] and RA [8, 20]. However, information related to undifferentiated spondyloarthropathies and AS is limited [21, 22, 31]. It is also the case for PsA [24].

In our series, the frequency of 25(OH)D deficiency (level <20 ng/ml) was higher in patients with RA than in the individuals from the non-CIRD control group. The present study raises several points of potential interest. First, the CARMA cohort constitutes the largest series of comparisons of 25(OH)D levels in three well-established CIRD. In addition, a cohort of individuals without CIRD was used for comparison. Second, we assessed patients who were periodically followed at rheumatology outpatient clinics. Nevertheless, it is important to emphasize that baseline levels of 25(OH)D in the control population were also low, due to the inclusion in this population of a high percentage of subjects with osteoarthritis and/or osteoporosis, who are also likely to have low baseline 25(OH)D levels.

Nowadays it is not clear whether vitamin D deficiency is the cause or effect of the inflammatory process. In this regard, in a model of acute phase response after surgery, plasma concentrations of 25(OH)D were found to decrease after elective knee arthroplasty [32]. Furthermore, several studies have found an inverse association between 25(OH)D levels and activity parameters of some CIRD, such as DAS28, swollen joints and HAQ in RA and BASFI and BASDAI in AS [17, 19, 21]. Although the results from our study do not fully support all these findings, in the multivariate analysis a marginally statistically significant association between 25(OH)D deficiency and ACPA in RA and BASFI in AS was found. It is worth noting that our patients with CIRD had decreased 25(OH)D levels despite the fact that a great majority had low activity at the time of inclusion. Patients with CIRD have less mobility and life outdoors, which would also contribute negatively to maintain adequate levels of vitamin D. Therefore, 25(OH)D deficiency in these patients may be explained by a dual mechanism. On the one hand, chronic diseases can predispose to 25(OH)D deficiency directly by decreasing synthesis or increasing vitamin D catabolism, and on the other hand, indirectly lowering sunlight exposure in phases of reduced mobility and ability to spend time outdoors in patients with worse functional status.

We feel that our results may be considered of potential interest in daily clinical practice, as our population encompassed individuals periodically followed at rheumatology outpatient clinics, many of whom are controlled under biological treatment.

Although a recent umbrella review of systematic reviews and meta-analyses of observational studies and randomized trials did not demonstrate that supplementation of vitamin D improves the health of the general population [33], we believe it is important to monitor and supplement vitamin D to patients with CIRD and vitamin D deficiency, regardless of whether the deficiency of vitamin D may or may not have a pathogenic role, or whether it is merely an epiphenomenon associated with inflammatory disease.

Our study has some limitations. First, a potential limitation of the study was that the non-CIRD subjects were not completely healthy, as a high percentage of individuals included had osteoarthritis, osteoporosis and/or other musculoskeletal diseases, which by themselves are associated with some risk of 25(OH)D deficiency. Another limitation of this study may be that the control group had more sunshine hours because many controls were recruited in the months of October and November, and several studies indicate that the level of 25(OH)D is the result of sun exposure in a period of time between 60 to 90 days prior to the 25(OH)D assessment [34], which in our study corresponded with the months of July and August. With respect to the variability of the vitamin D measurement among all participating centers, we performed a mixed model of logistic regression to reduce the variability in the method of assessment of 25(OH)D levels.

Finally, another limitation is the possible ecological fallacy that we may be committing to impute the average hours of sunshine from one province to every individual. As the CARMA study was designed to determine the causality of cardiovascular mortality in patients with CIRD, information on the length of time during which individual patients were exposed to sunshine was not collected. Therefore, and because sun exposure is a key factor in the blood levels of vitamin D, and patients were not recruited in the same period of the year and in the same geographical area of the country, we decided to collect aggregate information on sunshine hours provided by the SMA as an adjustment variable in the multivariate model.

## Conclusions

In summary, patients with RA followed at rheumatology outpatient clinics have high risk of 25(OH)D deficiency, in spite of presenting low-to-moderate disease activity due to tight control of the disease. In consequence, we believe that we must monitor the levels of vitamin D at baseline and during follow up, and supplement vitamin D if any deficiency is detected.

## Additional file

Additional file 1: Table S1. Distribution of the patients and controls according to the geographic area (region) and the month of the year of inclusion in the study. (DOC 70 kb)

## Abbreviations

25(OH) D: 25-hydroxyvitamin D
ACPA: anti-cyclic citrullinated peptide antibodies
ACR: American College of Rheumatology
AS: ankylosing spondylitis
BASDAI (0–10): Bath ankylosing spondylitis disease activity index
BASFI (0–10): Bath ankylosing spondylitis functional index
BMI: body mass index
CARMA: Cardiovascular in rheumatology project
CIRD: chronic inflammatory rheumatic diseases
CRP: C-reactive protein
DAS28-ESR: Disease activity score including 28 joints-erythrocyte sedimentation rate
HAQ (0–3): Health assessment questionnaire
HLA-B27: histocompatibility leucocyte antigen B27
IQR: interquartile range
OR: odds ratio
p25 − p75: 25^th^ to 75^th^ percentile
PsA: psoriatic arthritis
RA: rheumatoid arthritis
RF: rheumatoid factor SLE, systemic lupus erythematosus
SMA: Spanish Meteorological Agency
