# Supplementary material for: Vitamin D deficiency in chronic inflammatory rheumatic diseases: results of the cardiovascular in rheumatology [CARMA] study
Source: Arthritis Res Ther. 2015 Aug 14;17(1):211. doi: 10.1186/s13075-015-0704-4 (PMC4535672; doi:10.1186/s13075-015-0704-4)
Supplement: Additional file 1: Table S1. — Distribution of the patients and controls according to the geographic area (region) and the month of the year of inclusion in the study. (DOC 70 kb) [file 13075_2015_704_MOESM1_ESM.doc]

**Supplementary Table 1.** Distribution of the patients and controls according to the geographic area (region) and the month

of the year of inclusion in the study.

|  | Rheumatoid Arthritis | Ankylosing Spondylitis | Psoriatic Arthritis | Controls |
| --- | --- | --- | --- | --- |
| **Region (geographic area)** |  |  |  |  |
| Galicia, n (%) | 16 (2.1) | 17 (2.3) | 19 (2.6) | 17 (2.5) |
| Asturias, n (%) | 15 (1.9) | 15 (2) | 15 (2.1) | 12 (1.8) |
| Cantabria, n (%) | 21 (2.7) | 11 (1.5) | 14 (1.9) | 20 (3) |
| [Castile and Leon](http://en.wikipedia.org/wiki/Castile_and_León), n (%) | 36 (4.6) | 34 (4.6) | 30 (4.2) | 26 (3.9) |
| Basque Country, n (%) | 27 (3.5) | 27 (3.7) | 27 (3.7) | 21 (3.1) |
| Catalonia, n (%) | 144 (18.6) | 129 (17.5) | 128 (17.8) | 135 (20) |
| Aragon, n (%) | 40 (5.2) | 41 (5.6) | 41 (5.7) | 40 (6) |
| [Balearic Islands](http://en.wikipedia.org/wiki/Balearic_Islands), n (%) | 26 (3.4) | 18 (2.4) | 23 (3.2) | 17 (2.5) |
| [Valencian Community](http://en.wikipedia.org/wiki/Valencian_Community), n (%) | 47 (6.1) | 35 (4.7) | 36 (5.0) | 33 (4.9) |
| Murcia, n (%) | 18 (2.3) | 20 (2.7) | 18 (2.5) | 20 (3) |
| Madrid, n (%) | 185 (23.9) | 183 (24.8) | 186 (25.8) | 166 (25) |
| [Castile-La Mancha](http://en.wikipedia.org/wiki/Castilla-La_Mancha), n (%) | 55 (7.1) | 60 (8.1) | 48 (6.7) | 43 (6.4) |
| Extremadura, n (%) | 30 (3.9) | 30 (4.1) | 30 (4.2) | 30 (4.5) |
| Andalusia, n (%) | 73 (9.4) | 77 (10.4) | 67 (9.3) | 74 (11) |
| Canary Islands, n (%) | 42 (5.4) | 41 (5.6) | 39 (5.4) | 23 (3.4) |
| **Recruitment pattern** |  |  |  |  |
| July 2010, n (%) | 2 (0.2) | 1 (0.1) | 1 (0.1) |  |
| August 2010, n (%) | 3 (0.4) | 2 (0.3) | 2 (0.3) |  |
| September 2010, n (%) |  | 2 (0.3) | 1 (0.1) | 3 (0.4) |
| October 2010, n (%) |  | 1 (0.1) |  | 1 (0.1) |
| November 2010, n (%) | 11 (1.4) | 7 (0.9) | 3 (0.4) |  |
| December 2010, n (%) | 36 (4.6) | 23 (3.1) | 18 (2.5) | 2 (0.3) |
| January 2011, n (%) | 49 (6.3) | 25 (3.4) | 27 (3.7) | 4 (0.6) |
| February 2011, n (%) | 151 (19.5) | 82 (11.1) | 75 (10.4) | 18 (2.7) |
| March 2011, n (%) | 172 (22.2) | 139 (18.8) | 164 (22.7) | 34 (5.0) |
| April 2011, n (%) | 71 (9.2) | 100 (13.6) | 78 (10.8) | 34 (5.0) |
| May 2011, n (%) | 68 (8.8) | 73 (9.9) | 54 (7.5) | 17 (2.5) |
| June 2011, n (%) | 55 (7.1) | 66 (8.9) | 60 (8.3) | 34 (5.0) |
| July 2011, n (%) | 32 (4.1) | 53 (7.2) | 40 (5.5) | 18 (2.7) |
| August 2011, n (%) | 22 (2.8) | 29 (3.9) | 39 (5.4) | 40 (5.9) |
| September 2011, n (%) | 39 (5.0) | 26 (3.5) | 47 (6.5) | 31 (4.6) |
| October 2011, n (%) | 47 (6.1) | 1 (0.1) | 72 (10.0) | 146 (21.6) |
| November 2011, n (%) | 17 (0.2) | 7 (0.9) | 40 (5.5) | 279 (41.2) |
| December 2011, n (%) |  | 1 (0.1) |  | 9 (1.3) |
| January 2012, n (%) |  |  |  | 7 (1.0) |
